# Berberine Protects against NEFA-Induced Impairment of Mitochondrial Respiratory Chain Function and Insulin Signaling in Bovine Hepatocytes

**DOI:** 10.3390/ijms19061691

**Published:** 2018-06-06

**Authors:** Zhen Shi, Xiao-Bing Li, Zhi-Cheng Peng, Shou-Peng Fu, Chen-Xu Zhao, Xi-Liang Du, Zhi-Yuan Fang, Zhe Wang, Guo-Wen Liu, Xin-Wei Li

**Affiliations:** Key Laboratory of Zoonosis, Ministry of Education, College of Veterinary Medicine, Jilin University, Changchun 130062, China; shizhen16@mails.jlu.edu.cn (Z.S.); shawn.li@163.com (X.-B.L.); pengzc13@mails.jlu.edu.cn (Z.-C.P.); fushoupeng@jlu.edu.cn (S.-P.F.); zhaocx14@mails.jlu.edu.cn (C.-X.Z.); dxl16@mails.jlu.edu.cn (X.-L.D.); fangzy17@mails.jlu.edu.cn (Z.-Y.F.); wangzhe500518@sohu.com (Z.W.)

**Keywords:** dairy cows, fatty liver, Berberine, mitochondrial respiratory chain function, insulin signaling

## Abstract

Fatty liver is a major lipid metabolic disease in perinatal dairy cows and is characterized by high blood levels of non-esterified fatty acid (NEFA) and insulin resistance. Berberine (BBR) has been reported to improve insulin sensitivity in mice with hepatic steatosis. Mitochondrial dysfunction is considered a causal factor that induces insulin resistance. This study investigates the underlying mechanism and the beneficial effects of BBR on mitochondrial and insulin signaling in bovine hepatocytes. Revised quantitative insulin sensitivity check index (RQUICKI) of cows with fatty liver was significantly lower than that of healthy cows. Importantly, the Akt and GSK3β phosphorylation levels, protein levels of PGC-1α and four of the five representative subunits of oxidative phosphorylation (OXPHOS) were significantly decreased in cows with fatty liver using Western Blot analysis. In bovine hepatocytes, 1.2 mmol/L NEFA reduced insulin signaling and mitochondrial respiratory chain function, and 10 and 20 umol/L BBR restored these changes. Furthermore, activation of PGC-1α played the same beneficial effects of BBR on hepatocytes treated with NEFA. BBR treatment improves NEFA-impaired mitochondrial respiratory chain function and insulin signaling by increasing PGC-1α expression in hepatocytes, which provides a potential new strategy for the prevention and treatment of fatty liver in dairy cows.

## 1. Introduction

Fatty liver is a major lipid metabolic disorder of dairy cows in early lactation [1]. In dairy cows, fatty liver occurs primarily in the first 4 weeks after calving, when up to 50% of all dairy cows exhibit some accumulation of triglycerides (TG) in the liver [2,3], and is associated with decreased health status, well-being, productivity, and reproductive performance of cows [1]. The pathologic basis of fatty liver is negative energy balance (NEB) [4,5]. During the early lactation period, most dairy cows experienced a NEB caused by the decreased dry matter intake and increased demand for energy to support milk production [6]. This NEB initiates fat mobilization and a subsequent increase in non-esterified fatty acid (NEFA) blood concentration [7]. High levels of NEFA are re-esterified into TG and induce hepatic steatosis [8,9,10].

High levels of NEFA, a pathological factor for nonalcoholic fatty liver disease (NAFLD), demonstrate lipotoxicity and can impair hepatic mitochondrial function and insulin signaling [8,9,10]. Rector et al. [11] reported that mitochondrial dysfunction contributes to the development of insulin resistance and hepatic steatosis in obese rodent models. Furthermore, the maintenance of mitochondrial function and insulin sensitivity requires some regulators, such as peroxisome proliferator-activated receptor-γ coactivator (PGC)-1α, which plays a critical role in regulating mitochondrial biogenesis and the insulin signaling pathway [12,13]. Increasing evidence demonstrated that dairy cows with fatty liver displayed insulin resistance and high blood concentration of NEFA [4,7]. However, the effects of NEFA on the mitochondrial respiratory chain function and insulin signaling are not well characterized in cow hepatocytes.

**Figure 1:**
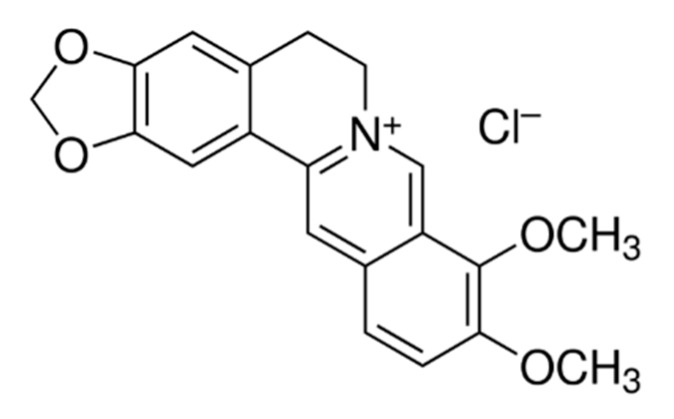
The chemical structure of berberine (BBR).

Berberine (BBR, Figure 1) is an isoquinoline alkaloid present in several plants used in traditional Chinese medicine. Multiple pharmacologic effects of BBR have been reported, including anti-inflammatory [14,15], anti-hypertensive [16], and anti-hepatic fibrosis [17], and there is no cytotoxic effect in healthy hepatocytes [18]. More importantly, BBR has a liver protective effect [19]. The beneficial effects of BBR on insulin sensitivity and hepatic steatosis show promise in the treatment of metabolic disorders, such as hyperlipidemia and diabetic rats [20,21]. Dairy cows with fatty liver displayed insulin resistance, high blood levels of NEFA and hepatic TG accumulation. The results obtained from mice demonstrated that mitochondrial dysfunction contributed to the development of insulin resistance [11]. Nevertheless, the hepatic mitochondrial respiratory chain function has not been evaluated to date in dairy cows with fatty liver. Therefore, the aim of this study is to (1) characterize the respiratory chain function in dairy cows with fatty liver; (2) investigate the effect of high levels of NEFA on the mitochondrial respiratory chain function and insulin signaling in cow hepatocytes; and (3) determine whether BBR can improve mitochondrial respiratory chain function and insulin signaling in bovine hepatocytes and in the underlying mechanism.

## 2. Results

### 2.1. Blood Biochemical Index and Liver Steatosis in Dairy Cows

**Figure 2:**
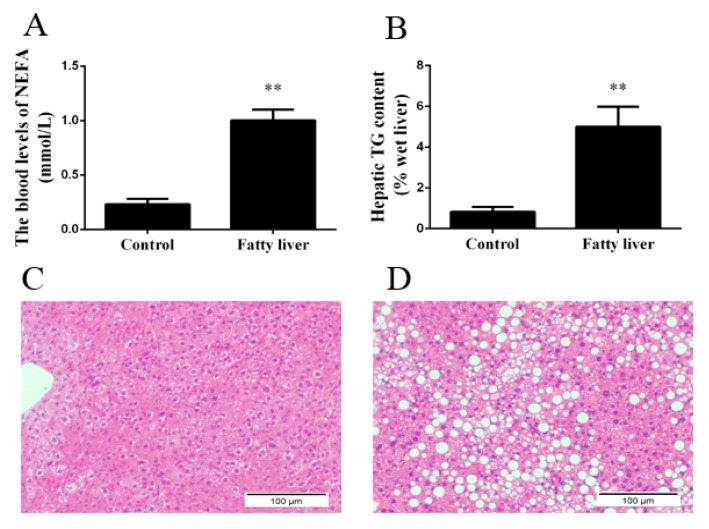
Dairy cows were classified into a healthy group and a fatty liver group. (**A**) The blood levels of NEFA; (**B**) Hepatic TG content; (**C**) H&E staining (×10) in liver sections in the healthy group; (**D**) H&E staining (×10) in liver sections in the fatty liver group. Quantified data are mean ± SD; ** *p* < 0.01 versus control group.

To assess the liver health of dairy cows, we detected the blood biochemical indicators. As shown in Figure 2A and Table 1, the blood levels of NEFA, insulin, alanine aminotransferase (ALT), aspartate aminotransferase (AST), and total bilirubin (TBIL) were significantly higher, but alkaline phosphatase (ALP) and glucose (GLU) were markedly lower in dairy cows with fatty liver than in healthy cows. These results indicate that dairy cows display negative energy balance and liver injury. Additionally, cows with fatty liver displayed hepatic TG accumulation (Figure 2B). Liver sections were stained with H&E to assess hepatic steatosis. The livers of healthy cows presented normal hepatocytic texture, and the liver cells exhibited homogenous cytoplasm and large spherical nuclei (Figure 2C). Livers isolated from cows with fatty liver exhibited severe steatosis, and the liver cells exhibited a lot of lipid vacuoles (Figure 2D).

### 2.2. Dairy Cows with Fatty Liver Displayed Impaired Hepatic Insulin Signaling and Mitochondrial Respiratory Chain Function

**Figure 3:**
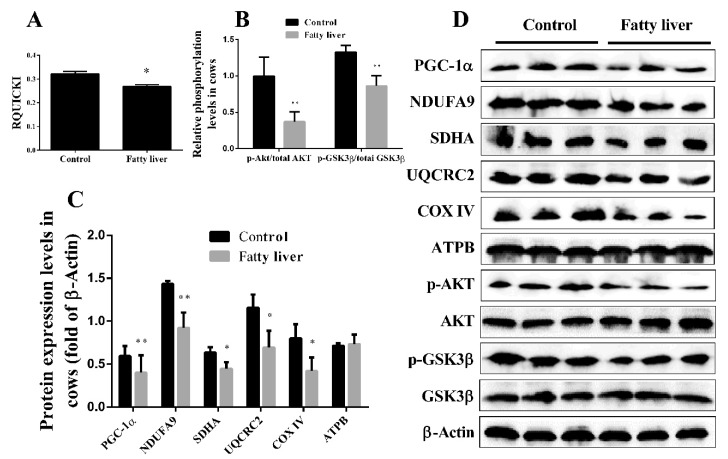
Dairy cows with fatty liver displayed impaired hepatic insulin signaling and mitochondrial respiratory chain function. (**A**) The value of revised quantitative insulin sensitivity check index (RQUICKI) in healthy and fatty liver cows; (**B**–**D**) Western blot analysis and quantification of key molecules of the insulin signaling pathway, PGC-1α, and five representative subunits of oxidative phosphorylation (OXPHOS) complexes in the liver of healthy and fatty liver cows. β-Actin served as an internal control. Quantified data are mean ± SD; * *p* < 0.05 versus control group; ** *p* < 0.01 versus control group.

Revised quantitative insulin sensitivity check index (RQUICKI) implies a valuation of the homeostatic energy balance and is based on plasma concentrations of glucose, insulin and free fatty acids. Holtenius and his colleague [22] demonstrated that RQUICKI could be used as an evaluation index of insulin sensibility in dairy cows. Accordingly, RQUICKI was calculated, and we found that RQUICKI was significantly higher in healthy cows than in the cows with fatty liver (Figure 3A). Although the evaluation of insulin resistance in dairy cows determined primarily by RQUICKI in previous studies is enough, in the present study, to further investigate the insulin sensitivity of cows with fatty liver, the hepatic insulin signaling pathway was examined. Compared with the healthy cows, the phosphorylation levels of Akt (phospho-Akt/Akt) and GSK3β (phospho-GSK3β/GSK3β) were significantly decreased in the cows with fatty liver (Figure 3B,D). Taken together, these results indicate that cows with fatty liver exhibit impaired hepatic insulin signaling.

Mitochondrial function is associated with insulin sensitivity. However, the hepatic mitochondrial respiratory chain function of cows with fatty liver was not evaluated in previous studies. The expression of five representative subunits for oxidative phosphorylation (OXPHOS) complexes (NDUFA9 (COI), SDHA (COII), UQCRC2 (COIII), COXIV (COIV) and ATPB (COV)) was usually used to evaluate the mitochondrial respiratory chain function. The protein expression levels of COI~IV were significantly lower in the cows with fatty liver than in the healthy cows (Figure 3C,D). Additionally, the expression level of ATPB was lower in the cows with fatty liver, but there was no significant difference. PGC-1α has been shown to play critical roles in regulating mitochondrial biogenesis, respiration, function and oxidative phenotype [13]. A previous study demonstrated that increasing the expression of PGC-1α could improve mitochondrial dysfunction and insulin resistance [13]. In this study, the protein expression of PGC-1α was significantly decreased in the cows with fatty liver than in healthy cows (Figure 3C,D). Taken together, these results indicate that cows with fatty liver exhibit impaired hepatic mitochondrial biogenesis and respiratory chain function.

### 2.3. BBR Treatment Improved the NEFA-Induced Lipid Accumulation in Bovine Hepatocytes

**Figure 4:**
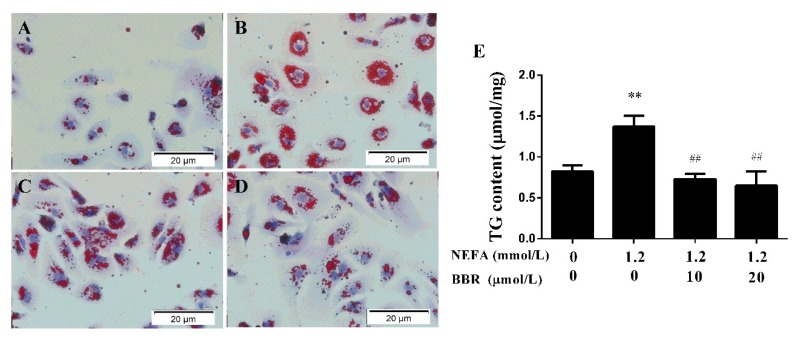
BBR decreased NEFA-induced TG accumulation in bovine hepatocytes. Oil red O staining (×40): (**A**) The normal bovine hepatocytes; (**B**) The bovine hepatocytes were pretreated with 1.2 mmol/L NEFA for 9 h; (**C**) After NEFA treatment, the hepatocytes were treated with 10 μmol/L BBR for another 24 h; (**D**) After NEFA treatment, the hepatocytes were treated with 20 μmol/L BBR for another 24 h; (**E**) BBR treatment significantly reduced the NEFA-induced increase in TG content. Quantified data are mean ± SD; ** *p* < 0.01 versus control group; ^##^ *p* < 0.01 versus NEFA group.

High levels of NEFA are the main pathologic factor in the development of fatty liver in dairy cows. To confirm whether high levels of NEFA can cause lipid accumulation, mitochondrial respiratory chain function and insulin signaling reduction in bovine hepatocytes, we administered the hepatocytes with high levels of NEFA. We then measured the TG content with a kit and stained the hepatocytes with an oil red O to determine lipid accumulation in hepatocytes. As shown in Figure 4, 1.2 mmol/L NEFA induced severe lipid accumulation, and 10 μmol/L BBR abolished this effect, with a 20 μmol/L abolishment effect being more pronounced. These results indicate that BBR can improve NEFA-induced lipid accumulation in a dose-dependent manner in bovine hepatocytes.

### 2.4. BBR Treatment Improved NEFA-Induced Mitochondrial Respiratory Chain Function and Insulin Signaling Damage by Increasing PGC-1α in Bovine Hepatocytes

**Figure 5:**
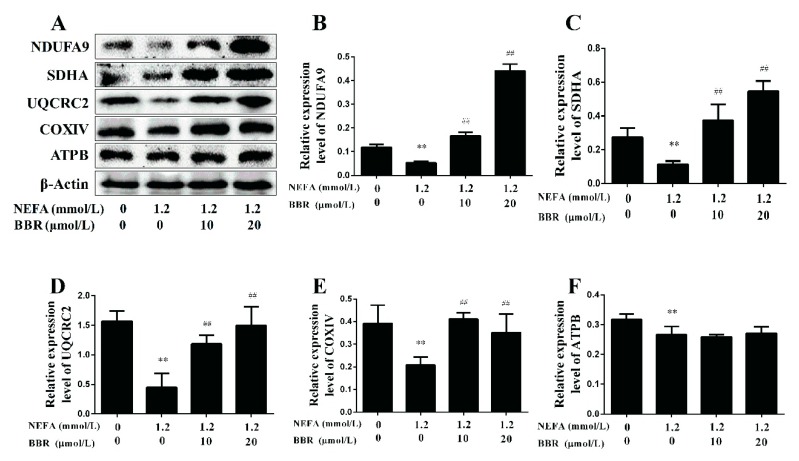
BBR improved the NEFA-induced mitochondrial respiratory chain function damage in bovine hepatocytes. Hepatocytes were assigned to four groups as follows: A control group, a 1.2 mmol/L NEFA treatment group, a 1.2 mmol/L NEFA and 10 μmol/L BBR treatment group, a 1.2 mmol/L NEFA and 20 μmol/L BBR treatment group. (**A**–**F**) Western blot analysis and quantification of five representative subunits of OXPHOS complexes, β-Actin served as an internal control. Quantified data are mean ± SD; ** *p* < 0.01 versus control group; ^##^ *p* < 0.01 versus NEFA group.

**Figure 6:**
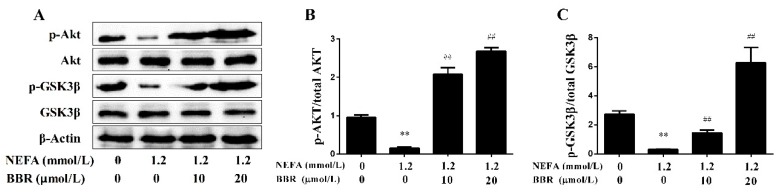
BBR improved the NEFA-induced insulin signaling damage in bovine hepatocytes. Hepatocyte treatment is described as in Figure 5. (**A**–**C**) Western blot analysis and quantification of key molecules of the insulin signaling pathway, β-Actin served as an internal control. Quantified data are mean ± SD; ** *p* < 0.01 versus control group; ^##^ *p* < 0.01 versus NEFA group.

To clarify the effects of NEFA on the mitochondrion in primary hepatocytes, the protein expression levels of five representative subunits of OXPHOS complexes were detected using a Western blot. As shown in Figure 5, the protein expression levels of COI~IV were significantly lower in the NEFA treatment group than in the control group, and the protein expression level of COV had no change between groups. These results indicate that high levels of NEFA can inhibit the expression of OXPHOS subunits and reduce mitochondrial respiratory chain function in hepatocytes. Moreover, the effect of NEFA on the insulin signaling pathway was also detected. The phosphorylation levels of Akt and GSK3β were significantly decreased in the NEFA treatment group (Figure 6). These results suggest that high levels of NEFA treatment markedly impair the insulin signaling pathway in hepatocytes.

A previous study demonstrated that BBR had beneficial effects on the metabolic syndrome in humans [23]. As shown in Figure 5, BBR treatment upregulated the protein expression levels of COI~IV. Furthermore, the phosphorylation levels of Akt and GSK3β were significantly increased in the BBR+NEFA group compared with the NEFA group (Figure 6). Importantly, the beneficial effects of BBR on the mitochondrial respiratory chain function and insulin signaling were presented in a dose-dependent manner. These results indicate that BBR can markedly improve the impairment of mitochondrial respiratory chain function and insulin signaling induced by NEFA in bovine hepatocytes.

### 2.5. PGC-1α Mediated the Improvement Effect of BBR on NEFA-Induced Mitochondrial Respiratory Chain Function and Insulin Signaling Damage in Bovine Hepatocytes

**Figure 7:**
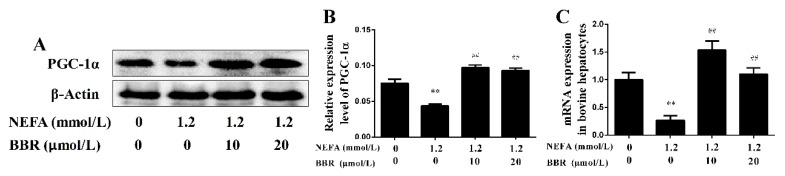
Effects of BBR on the expression of PGC-1α in bovine hepatocytes. Hepatocyte treatment is described in Figure 5. (**A**,**B**) Western blot analysis and quantification of PGC-1α, β-Actin served as an internal control; (**C**) PGC-1α mRNA expression level changed in different groups. Quantified data are mean ± SD; ** *p* < 0.01 versus control group; ^##^ *p* < 0.01 versus NEFA group.

**Figure 8:**
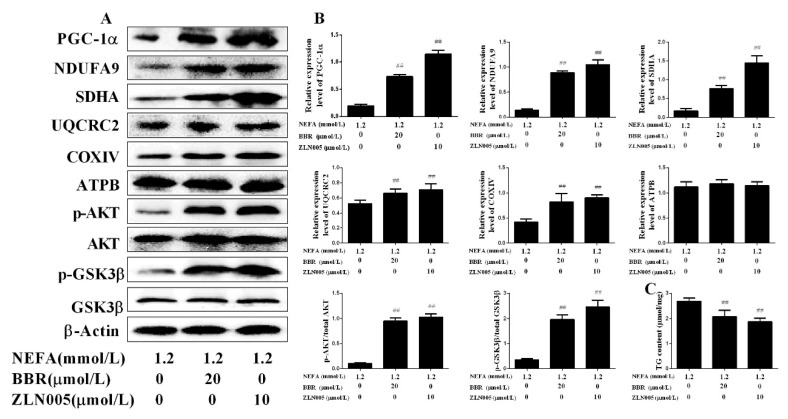
Activation of PGC-1α increased the beneficial effects of BBR on mitochondrial respiratory chain function and insulin signaling damage induced by NEFA. Hepatocytes were assigned to 3 groups as follows: A 1.2 mmol/L NEFA group, a 1.2 mmol/L NEFA + 20 μmol/L BBR treatment group, and a 1.2 mmol/L NEFA + 10 μmol/L ZLN005 treatment group. (**A**,**B**) Western blot analysis and quantification of key molecules of the insulin signaling pathway, PGC-1α, and five representative subunits of OXPHOS complexes, and β-Actin served as an internal control; (**C**) TG content in bovine hepatocytes. Quantified data are mean ± SD; ^##^ *p* < 0.01 versus NEFA group.

In bovine hepatocytes, NEFA treatment also significantly down-regulated the mRNA and protein levels of PGC-1α. Importantly, BBR treatment markedly improved the inhibition effect of NEFA on PGC-1α expression (Figure 7). These results indicate that the beneficial effect of BBR on mitochondrial respiratory chain function and insulin signaling damage induced by NEFA may occur by increasing PGC-1α expression. To further demonstrate this speculation, bovine hepatocytes were treated with ZLN005 (a PGC-1α agonist) after NEFA treatment. As shown in Figure 8A,B, the protein expression levels of COI~IV and the phosphorylation levels of Akt and GSK3β in the NEFA+ZLN005 group were significantly higher than in the NEFA group, suggesting that activation of PGC-1α could improve the mitochondrial respiratory chain function and insulin signaling damage induced by NEFA. Importantly, the trend of TG content in different groups was consistent with the above results (Figure 8C). These results indicate that PGC-1α mediates the improvement effect of BBR on NEFA-induced mitochondrial respiratory chain function and insulin signaling damage in bovine hepatocytes.

## 3. Discussion

Fatty liver is a major metabolic disorder in perinatal dairy cows. Dairy cows with fatty liver displayed insulin resistance, which in turn further promoted the development of fatty liver [4,7]. In a previous study, the evaluation of insulin resistance was primarily determined by RQUICKI [22]. Our data showed that the RQUICKI of cows with fatty liver was significantly lower than healthy cows, which suggested that dairy cows with fatty liver exhibited insulin resistance. However, the detection of the hepatic insulin pathway sensibility could provide more valuable information to evaluate the insulin resistance in dairy cows with fatty liver. In this study, hepatic samples were collected, and the hepatic insulin signaling pathway was detected. The phosphorylation levels of Akt and GSK3β were significantly decreased in the cows with fatty liver, which further indicated that cows with fatty liver exhibited impaired insulin signaling.

Mitochondria are the primary organelles of fatty acid oxidation and cellular ATP production, which play a crucial role in the regulation of energy metabolism balance. The results from NAFLD patients and high-fat diet mice demonstrated that mitochondrial dysfunction was associated with the development of insulin resistance and fatty liver [11,24]. Nevertheless, previous studies did not evaluate the function of mitochondria in dairy cows with fatty liver. Under these conditions, fresh liver biopsies were performed to determine the potential hepatic cellular and molecular changes that relate to mitochondrial function in cows. As we know, PGC-1α and OXPHOS, regulates mitochondrial biogenesis and the terminal process of eukaryotic mitochondrial respiratory chain, respectively. Therefore, we detected PGC-1α and OXPHOS to evaluate the status of the mitochondria. The results showed that protein expression levels of PGC-1α and OXPHOS complexes subunit COI~IV were significantly decreased in the livers of cows with fatty liver. These results demonstrate that cows with fatty liver exhibit a damage in mitochondrial biogenesis and respiratory chain function, which further indicates hepatic mitochondrial dysfunction has largely occurred. Furthermore, the results from mice with nonalcoholic fatty liver disease demonstrated that mitochondrial dysfunction was associated with the induction of insulin resistance [25,26,27]. Therefore, the hepatic insulin resistance might be partly induced by mitochondrial dysfunction in dairy cows with fatty liver, which is consistent with previous studies [11]. Insulin signaling in the liver is critical in regulating glucose homeostasis and maintaining normal hepatic function [28]. Impaired insulin signaling further increased the hepatic TG accumulation and steatosis in dairy cows.

The blood concentration of NEFA was significantly increased in dairy cows with fatty liver. High levels of NEFA could cause cell lipotoxicity and induce oxidative stress, apoptosis and inflammation in cow hepatocytes [29,30]. In this study, the results of cow hepatocytes demonstrated that NEFA treatment could induce impairment of mitochondrial respiratory chain function and insulin signaling. Additionally, NEFA treatment could significantly inhibit the expression of PGC-1α, an important regulator of mitochondrial function. These results indicate that high levels of NEFA induce impairment of mitochondrial respiratory chain function and insulin signaling in the livers of dairy cows with fatty liver.

BBR has been used as a therapy to treat a variety of metabolic diseases in Korea, China, and possibly other Asian countries that practice the use of traditional medicines [31]. Lee et al. [32] reported that BBR activated AMP-activated protein kinase with beneficial metabolic effects in diabetic and insulin-resistant states. Lou et al. [33] demonstrated that BBR treatment increased the phosphorylation of Akt and then improved insulin sensitivity in PA-stimulated hepatocytes. Furthermore, data from rats demonstrated that BBR protected against high-fat diet-induced mitochondrial dysfunction in muscle [21]. Teodoro et al. [31] reported that BBR could revert hepatic mitochondrial dysfunction in high-fat diet rats. These studies indicated that BBR could improve insulin resistance and mitochondrial dysfunction in rodents. Nevertheless, there was no study to report whether BBR could improve NEFA-induced mitochondrial and insulin signaling damage in bovine hepatocytes. In this study, we choose 10 and 20 μmol/L BBR to administrate bovine hepatocytes according to the previous study [17,33,34]. In a previous study, Lou et al. reported that 10 μmol/L BBR increased the phosphorylation of Akt in HepG2 cells [33], Song et al. reported that 10 μg/mL (>40 μmol/L) BBR increased the phosphorylation of AKT and GSK3β in B16F10 melanoma cells [35], and Gomes et al. reported that 5 μmol/L BBR increased mitochondrial membrane potential in C2C12 cells [21]. Consistent with previous results, our data also showed that 10 or 20 μmol/L BBR increased the phosphorylation levels of Akt and GSK3β and the protein expression levels of OXPHOS complexes I, II, III and IV in NEFA-treated bovine hepatocytes. The results indicate that these concentrations of BBR play a beneficial role in insulin signaling and mitochondrial respiratory chain function in bovine hepatocytes. Importantly, we also found that BBR treatment could reverse the inhibition effect of NEFA on the PGC-1α. Therefore, we speculated that BBR might improve mitochondrial respiratory chain function and insulin signaling by targeting PGC-1α. To further demonstrate this speculation, bovine hepatocytes were treated with ZLN005 after NEFA treatment. As we expected, ZLN005 significantly increased the expression of PGC-1α and improved mitochondrial respiratory chain function, insulin signaling and intracellular TG accumulation in hepatocytes. These data indicate that the protective effect of BBR treatment on improving the NEFA-induced impairment of mitochondrial respiratory chain function and insulin signaling, at least in part, were mediated by PGC-1α signaling in bovine hepatocytes.

Fatty liver in dairy cows is divided into mild (1–5% liver TG on wet weight basis), moderate (5–10% liver TG) and severe (above 10% liver TG) fatty liver [1]. The clinical investigation showed that incidence of fatty liver was mainly mild and moderate fatty liver in dairy cows. Importantly, in dairy cows, long-term suffering from fatty liver is associated with decreased milk production and longer calving intervals, which greatly increases veterinary costs and reduces the economic benefits of the dairy industry [7]. Our data demonstrated that BBR could significantly improve the mitochondrial respiratory chain function and insulin signaling damage induced by NEFA through PGC-1α in bovine hepatocytes. Furthermore, the results from high-fat diet mice or humans with Type 2 diabetes further supported the beneficial effects of BBR on insulin resistance [31,32]. Therefore, BBR could be applied to the prevention of fatty liver, as a feed additive, in the transition period of dairy cows.

## 4. Materials and Methods

### 4.1. Animals

Eight healthy Holstein cows and eight cows with fatty liver (in the second lactation) with similar body condition scores (BCSs) in each group were selected from a 1000 cow ecology dairy farm located in Changchun City, Jilin Province, China (2016 clinical trial [2016-126], 6 December 2016). All cows were subjected to routine physical examination. Eight cows with fatty liver were selected based on blood levels of NEFA and liver TG content (% wet liver), which are considered the gold standards for fatty liver diagnosis. Blood samples were extracted from the jugular vein and centrifuged at 1200× *g* for 15 min to obtain a serum. Hepatic samples were collected by an experienced veterinarian using a liver puncture needle between the cows’ 12th and 13th ribs. The Ethics Committee on the Use and Care of Animals at Jilin University approved the study protocol (Changchun, China). During the experimental work, the cows were housed in a climate-controlled barn in individual tie stalls to reduce environmental effects.

### 4.2. Determination of Blood Parameters

NEFA and glucose (GLU) levels were measured using standardized kits purchased from Gcell (Beijing Strong Biotechnologies, Beijing, China) and a Celercare^®^ V2 automatic biochemical analyzer (MNCHIP Technologies, Tianjin, China). Insulin concentration was detected by an enzyme-linked immunosorbent assay (ELISA) kit according to the manufacturer’s protocol (JianCheng, Nanjing, China). The concentrations of alanine aminotransferase (ALT), aspartate transaminase (AST), alkaline phosphatase (ALP), and total bilirubin (TBIL) were measured by using commercial kits (JianCheng). RQUICKI is calculated based on the blood plasma concentrations of glucose (Gb, mg/dL), insulin (Ib, μU/mL) and free fatty acids (FFAb, mmol/L); b denotes basal values. The formula was first described by Perseghin et al. [36] in the following manner: RQUICKI = 1/[log (Gb) + log (Ib) + log (FFAb)].

### 4.3. Bovine Hepatocyte Isolation and Culture

The study protocol was approved by the Ethics Committee on the Use and Care of Animals, Jilin University (Changchun, China). The hepatocytes were isolated by the collagenase IV perfusion method as described by previous studies [6,37]. The caudate lobe was perfused with perfusion solution until the liquid became clear. Then, the liver was perfused with collagenase IV solution to dissociate liver tissue structure until the liquid became muddy. Basic medium (4 °C) containing 0.2% bovine serum albumin (BSA) was added into the flat plate to terminate the digestion. The liver was cut open, and the liver capsule, blood vessels, fat, and connective tissue were removed. The liver suspension was filtered sequentially with 100 mesh (150 µm), 200 mesh (75 µm), and 400 mesh (37.5 µm) cell sieves. The hepatocyte suspension was washed twice with the basic medium. The cell viability was detected using the Trypan blue dye exclusion method, and the percentage of viable cells was nearly 99%. The hepatocyte suspension (1 × 10^6^ cells/mL) was seeded into a six-well tissue culture plate (2 mL per well) using an adherent medium and incubated at 37 °C in 5% CO_2_ and saturated humidity. After 4 h of incubation, the medium was replaced with a growth medium every 24 h. The perfusion solution, digestion solution, adherent medium and growth medium were prepared as in our previous study [6]. The cells were starved of serum overnight before treatment. The hepatocytes were treated with NEFA for 9 h, ZLN005 (MedChem Express, NJ, USA) for 24 h, and different levels of berberine (Sigma-Aldrich, St. Louis, MO, USA) for 24h. The detailed cell treatment and grouping are shown in the figure legend. The NEFA used in this study were based on serum NEFA concentrations in dairy cows with fatty liver [8]. The stock NEFA (52.7 mmol/L) solution contained oleic acid (22.9 mmol/L), linoleic acid (2.6 mmol/L), palmitic acid (16.8 mmol/L), stearic acid (7.6 mmol/L), and paltoleic acid (2.8 mmol/L) (Sigma-Aldrich), and the pH of the NEFA solution was adjusted to 7.2 using hydrochloric acid (1 mol/L).

### 4.4. H&E Staining of Hepatic Tissue and Oil Red O Staining of Cow Hepatocytes

Hepatic tissue was collected by liver biopsy using a liver puncture needle. Liver tissues were fixed in 4% paraformaldehyde, embedded in paraffin, sliced, and stained with hematoxylin and eosin (H&E). Hepatocytes from different administration were washed twice with phosphate buffer saline (PBS), 10% formaldehyde for 15 min, washed twice with PBS, stained with filtered oil red (oil red: water = 3:2) for 30 min, washed twice with PBS, stained with filtered hematoxylin for 5 min, washed with PBS for 7 min, and finally underwent microscopic observation with a glycerin film.

### 4.5. Determination of Hepatic and Cellular TG

Liver tissue was broken down using Micro Tissue Grinders (Tiangen Biotech, Beijing, China) with an extraction buffer (Beyotime Biotech, Haimen, China). The tissue suspension was centrifuged at 3000× *g* for 10 min to obtain supernatant, which was used to detect the TG content using a tissue triglyceride assay kit (JianCheng), following the manufacturer’s instructions. Bovine hepatocytes were collected from the 6-well plates after administration. The cells were lysed using a lysis buffer (Sangon Biotech, Shanghai, China) in an ice bath. The lysate was centrifuged at 12,000× *g* at 4 °C for 5 min, and the supernatant was collected for triglyceride analysis using the tissue triglyceride assay kit (JianCheng), according to the manufacturer’s protocol. Total protein concentration was estimated by the BCA method (Applygen, Beijing, China) and performed according to the manufacturer’s instructions. Hepatic and cellular TG were normalized to total protein contents.

### 4.6. RNA Extraction and Real-Time Quantitative PCR (qRT-PCR)

Total hepatic and cellular RNA were extracted using a TRIzol kit (TaKaRa, Dalian, China) according to the manufacturer’s instructions. Five microliters of RNA solution was used to detect its concentration using a Gene Quant II RNA/DNA Calculator (Pharmacia Biotech, Cambridge, UK). Furthermore, the RNA quality was determined by electrophoresis on 1% agarose gel. The RNA concentration in all samples was diluted to the same concentration using diethylpyrocarbonate water. Approximately 5 μg RNA in each sample was reverse-transcribed to cDNA in 20 μL functions using a reverse transcription kit (TaKaRa), according to the supplier’s protocol. The gene-specific primers were designed using Primer 5.0 (PE Applied Biosystems, Foster, CA, USA). The primer sequences for target genes were as follows: PGC-1α, forward 5′-CCCGTGCTACCTGAGAGAGA-3′ and reverse 5′-CTTGACTGGGATGACCGAAG-3′; β-Actin 5′-GCCCTGAGGCTCTCTTCCA-3′ and reverse 5′-GCGGATGTCGACGTCACA-3′. The mRNA expression levels were evaluated by qRT-PCR using a 7500 Real-Time PCR System (Applied Biosystems, Foster, CA, USA) and a SYBR green plus reagent kit (Roche, Norwalk, CT, USA). The relative expression of genes was calculated by 2^−∆∆*C*t^ and was normalized to β-Actin abundance.

### 4.7. Western Blotting

Western blotting assays were performed as described [38,39]. The liver and hepatocyte protein were extracted using a protein extraction kit (Sangon Biotech, Shanghai, China) according to the manufacturer’s instructions. The protein concentration was measured using protein assay reagent (Sangon Biotech). The target proteins were separated in polyacrylamide gels and electro-transferred onto polyvinylidene difluoride (PVDF) membranes. The membranes were blocked in a 3% BSA in 0.1% Tris-buffered saline and Tween (TBST) buffer for 4 h. The membranes were hybridized with antibodies specific for PGC-1α (1:1000), Akt (1:1000), GSK-3β (1:1000), P-Akt (1:1000), P-GSK-3β (1:1000), NDUFA9 (1:1000), SDHA (1:1000), UQCRC2 (1:1000), COXIV (1:1000), ATPB (1:1000) (Abcam, USA) and β-Actin (1:5000) (ZSGB-BIO, Beijing, China) overnight at 4 °C. The PVDF membranes were incubated with appropriate peroxidase-conjugated secondary antibodies for 45 min. Immunoreactive bands were detected with enhanced chemiluminescence solution (Beyotime Biotech). The blots were normalized to β-Actin and quantified via densitometry using Image-Pro Plus 6.0 (Media Cybermetics, Rockville, MD, USA). Experiments were carried out in triplicate.

### 4.8. Statistical Analysis

All experiments were conducted in 3 separate cell preparations from 3 calves using at least 3 replicates per treatment. The results are expressed as the mean ± standard deviation (SD). The data were analyzed by independent sample *T*-test or a one-way analysis of variance (ANOVA) followed by Duncan’s multiple range test (SPSS 19.0 software; SPSS Inc., Chicago, IL, USA). A *p*-value of less than 0.05 was considered significant, and values less than 0.01 were considered markedly significant.

## 5. Conclusions

Our study revealed that dairy cows with fatty liver displayed a damage to mitochondrial respiratory chain function and insulin signaling. High levels of NEFA could impair mitochondrial respiratory chain function and insulin signaling in bovine hepatocytes. Importantly, BBR treatment markedly improved impairment of mitochondrial respiratory chain function and insulin signaling induced by NEFA via PGC-1α in bovine hepatocytes, which provides a potential new therapeutic strategy for the prevention and treatment of fatty liver, especially for mild and moderate fatty liver, in dairy cows.

## Figures and Tables

**Table 1:** Blood biochemical index of the healthy and fatty liver cows.

Blood Biochemical Index	Healthy Cows	Cows with Fatty Liver	*p* Value
Body weight (kg)	536.8 ± 19.1	541.3 ± 18.2	0.779
INSULIN (mU/L)	1.45 ± 0.12	1.96 ± 0.16 *	0.011
GLU (mmol/L)	3.26 ± 0.44	2.09 ± 0.07 *	0.041
ALT (U/L)	14.79 ± 1.42	23.80 ± 1.29 **	0.001
AST (U/L)	80.79 ± 15.67	170.97 ± 9.25 **	0.001
AST/ALT	5.44 ± 0.68	7.18 ± 0.11 *	0.044
ALP (U/L)	67.5 ± 3.31	44.5 ± 4.35 **	0.002
TBIL (μmol/L)	2.37 ± 0.10	6.09 ± 0.78 **	0.001

Quantified data are mean ± SD; * *p* < 0.05 versus healthy cows; ** *p* < 0.01 versus healthy cows.
